# Variables associated with pulmonary hypertension screened by echocardiography in chronic myeloid leukemia patients on dasatinib therapy

**DOI:** 10.3389/fcvm.2022.960531

**Published:** 2022-08-09

**Authors:** Wenying Jin, Sen Yang, Chao Yu, Tiangang Zhu, Qian Jiang

**Affiliations:** ^1^Department of Cardiology, Beijing Key Laboratory of Early Prediction and Intervention of Acute Myocardial Infarction, Center for Cardiovascular Translational Research, Pekin University People’s Hospital, Beijing, China; ^2^National Clinical Research Center for Hematologic Disease, Beijing Key Laboratory of Hematopoietic Stem Cell Transplantation, Peking University People’s Hospital, Institute of Hematology, Beijing, China; ^3^Collaborative Innovation Center of Hematology, Soochow University, Suzhou, China

**Keywords:** tyrosine kinase inhibitor, leukemia, hypertension, pulmonary, myelogenous, chronic, *BCR:ABL1* positive

## Abstract

**Background:**

Pulmonary hypertension (PH) is a rare but life-threatening adverse event (AE) of dasatinib, but the associated variables are not clear. This study aimed to explore the variables associated with PH by echocardiography in patients with chronic myeloid leukemia in the chronic phase (CML-CP) receiving dasatinib therapy.

**Methods:**

Echocardiography was performed to estimate the probability of PH and pulmonary artery systolic pressure (PASP). Binary logistic analysis and Fine–Gray hazard model were used to identify the variables associated with PH by using cross-sectional and longitudinal data.

**Results:**

Among the 243 patients in the cross-sectional dataset, with a median dasatinib therapy duration of 27 months, 30 (12.3%) were classified as having a high probability of PH. Increasing age (OR = 1.7, *p* = 0.002; OR = 1.5, *p* = 0.003) and pericardial effusion (OR = 4.3, *p* = 0.004; OR = 3.2, *p* = 0.014) were significantly associated with a high probability of PH and PASP ≥ 40 mmHg, respectively. Among the 161 patients in the longitudinal dataset, the 3-year cumulative incidences of a high probability of PH and PASP ≥ 40 mmHg were 9.3% and 22.1%, respectively. Pericardial effusion (HR = 3.8, *p* = 0.005) and cardiopulmonary comorbidities (HR = 3.2, *p* = 0.021) were significantly associated with a high probability of PH; increasing age (HR = 1.5, *p* < 0.001) and dasatinib as ≥ 3rd-line therapy (*p* = 0.032; 2nd-line *vs.* 1st-line, HR = 2.0, *p* = 0.200; ≥ 3rd-line *vs.* 1st-line, HR = 3.4, *p* = 0.047) were significantly associated with PASP ≥ 40 mmHg.

**Conclusion:**

Increasing age, pericardial effusion, cardiopulmonary comorbidities, and dasatinib as ≥ 3rd-line TKI therapy were associated with PH in the patients with CML-CP on dasatinib therapy.

## Introduction

Pulmonary hypertension (PH) is a rare but life-threatening adverse event (AE) of dasatinib in patients with chronic myeloid leukemia (CML), which may result in poor outcomes and lead to right ventricular pressure overload, hypertrophy, dilation, and, ultimately, right heart failure (1). The incidence of dasatinib-related PH is 0.45–5% in symptomatic patients confirmed by right-heart catheterization (RHC) or with echocardiography screening (2, 3). Unlike other types of PH, such as idiopathic pulmonary arterial hypertension, heritable pulmonary arterial hypertension, or PH caused by anorectic agents that are progressive and irreversible, the symptoms and hemodynamics of dasatinib-related PH may improve in most patients after discontinuing dasatinib therapy. Dasatinib-related PH may be asymptomatic in the early stages of the disease and may eventually cause severe consequences (2, 4, 5). Previous studies have reported an increase in brain natriuretic peptide levels and that older age is associated with an elevated tricuspid regurgitation peak gradient and occurrence of dasatinib-related PH (6). However, such data are limited. Currently, RHC is the gold standard for PH diagnosis, and non-invasive transthoracic echocardiography is the most recommended screening method (1). Therefore, we designed a study to explore the prevalence and cumulative incidence of PH and its associated variables using echocardiography in patients with CML in the chronic phase (CML-CP) on dasatinib therapy.

## Materials and methods

### Study population

From April 2018 to April 2019, consecutive patients with CML-CP who were on dasatinib therapy at Peking University People’s Hospital were screened using echocardiography, electrocardiography, and chest radiography; this patient group was the cross-sectional cohort. Data of patients who had consecutive echocardiographic follow-up before and during dasatinib therapy from April 2008 to September 2021 were reviewed; this patient group was the longitudinal cohort. Demographic and clinical information and medical history were obtained from the medical records. Patients with a history of congenital heart disease, pulmonary embolism, connective tissue disease, or congestive heart failure were excluded. This study was approved by the local ethics committee, and informed consent was obtained from all patients.

### Diagnosis, monitoring, and responses

Diagnosis, monitoring, and therapy responses were performed according to the European LeukemiaNet recommendations (7–9). The EUTOS long-term survival score was used to assign a prognosis risk cohort (10). Bone marrow cytogenetic analyses were performed by G-banding. Blood *BCR:ABL1* transcript levels during therapy were analyzed by quantitative real-time polymerase chain reaction with *ABL1* as the control and converted to international scales (*BCR:ABL1^IS^*) using our laboratory-specific conversion factor of 0.65, which was validated by the Institute of Medical and Veterinary Science International Reference Laboratory (11, 12).

Chest X-ray was performed to monitor the occurrence of pleural effusion every 6 months or when the patients experienced the symptoms such as short of breath. Complete hematologic response was defined as a WBC concentration of <10 × 10E + 9/L, platelet concentration of <450 × 10E + 9/L, no immature myeloid cells in the blood, blood basophil level of <5%, and no extramedullary leukemia for ≥4 weeks. Complete cytogenetic response was defined as no Ph-chromsome cells in ≥20 bone marrow metaphases. Major molecular response was defined as *BCR:ABL1^IS^* ≤ 0.1%, Molecular response 4 (MR4) or deep molecular response as *BCR:ABL1^IS^* ≤ 0.01%, and MR4.5 as *BCR:ABL1^IS^* ≤ 0.0032% (7–9).

### Echocardiographic examination

Transthoracic echocardiographic examinations were performed in all patients using a GE Vivd E9 (GE Vingmed, Horten, Norway) or ACUSON SC2000 (Siemens, Munich, Germany) ultrasound system with a 2.5–3.5 MHz phased-array transducer. Standard two-dimensional echocardiography with Doppler examination was performed, and measurements were obtained according to the guidelines of the American Society of Echocardiography (13). Pericardial effusion was evaluated using simple semiquantitative echocardiographic assessment and classified according to its diastolic depth as mild (<10 mm), moderate (10–20 mm), or large (>20 mm) (14). The researchers were blinded to the clinical information.

### Diagnostic criteria of pulmonary hypertension

The 2015 European Society of Cardiology guideline recommended the echocardiographic PH probability classification to be based on the tricuspid regurgitation velocity (TRV) and additional PH signs as suggested by the guideline (1). Patients were classified as having “low” (TRV ≤ 2.8 m/s without additional PH signs), “intermediate” (TRV ≤ 2.8 m/s with additional PH signs or TRV 2.9–3.4 m/s without additional PH signs), or “high” PH probability (TRV 2.9–3.4 m/s with additional PH signs, or TRV > 3.4 m/s). Pulmonary artery systolic pressure (PASP) was determined by tricuspid regurgitation peak gradient using the simplified Bernoulli equation combined with an estimate of the right atrial pressure (RAP). PASP was calculated using the following equation: PASP = 4(V)^2^ + RAP, where V is the peak velocity (in m/s) of the tricuspid valve regurgitation jet. RAP was estimated based on the diameter and respiratory variation in the inferior vena cava. PASP ≥ 40 mmHg was considered abnormal and might be a sign of PH according to the expert consensus of the American College of Cardiology Foundation and American Heart Association (15).

### Statistical analysis

Continuous variables are expressed as median (range) or mean ± SD, and categorical variables are expressed as absolute numbers (percentages). Descriptive statistical analysis was used for the demographic characteristics of the patients. Inter-group comparisons of categorical variables were analyzed using Pearson’s chi-square test, and continuous variables were analyzed using the Mann–Whitney U test. Variables with univariate analysis p < 0.2 were included in the binary logistic regression model for multivariate analysis.

Pulmonary artery systolic pressure during the follow-up period was analyzed by paired Wilcoxon test and compared with the baseline level. The cumulative incidence of high probability of PH was calculated by a competing risk model using the Fine–Gray test considering competing events defined as switching to another tyrosine kinase inhibitor (TKI), chemotherapy, a transplant, or death, and data were censored at the last follow-up or at consent withdrawal.

Propensity score matching (PSM) was performed to adjust for differences in characteristics between patients receiving dasatinib doses of <100 mg/d or ≥100 mg/d in cross-sectional data and evaluated using the standardized absolute mean difference (SAMD). An SAMD < 0.02 was considered adequate balance.

All *p*-values were two-tailed, and statistical significance was set at *p* < 0.05. All statistical analyses were performed using SPSS (version 26.0; SPSS, Chicago, IL, United States) and R version 4.0.2 (R Core Team, Vienna, Austria).

## Results

### Patient characteristics

From April 2018 to April 2019, the data of 243 consecutive patients with CML-CP who visited Peking University People’s Hospital and were receiving dasatinib therapy were screened by echocardiography to establish a cross-sectional dataset (Table 1). The median age of the patients was 45 years (range, 21–78 years). 147 (61%) patients were male. The median duration of CML before dasatinib treatment was 28 months (range, 0–248 months), and the median dasatinib treatment duration was 22 months (range, 3–130 months). 223 (92%) patients received dasatinib as ≥2nd-line of therapy. 66 (27%) patients were treated with dasatinib at a current dosage of <100 mg/d.

**TABLE 1:** Patients’ characteristics.

	Cross-sectional cohort *N* = 243	Follow-up cohort *N* = 161
Male, n(%)	147(61%)	100(62%)
Age, y, median(range)	45(21–78)	46(11–79)
Cardiopulmonary diseases, n(%)	25(10%)	55(34%)
Smoke, n(%)	37 (15%)	37(23%)
Disease duration before dasatinib–therapy, m, median(range)	28(0–248)	10(0–231)
Dasatinib-therapy duration, m, median (range)	22(3–130)	24(3–156)
Dasatinib-therapy used as, n(%)		
First - line	20(8%)	33(21%)
Second - line	161(66%)	113(70%)
Third or fourth-line	62(26%)	15(9%)
Dasatinib dosage* < 100 mg/d, n(%)	66(27%)	52(32%)
Pericardial effusion, n(%)	23(10%)	41(25%)
Pleural effusion, n(%)	69(28%)	23(14%)
Response to dasatinib-therapy, n(%)		
<CCyR	48(20%)	33(21%)
CCyR	38(16%)	33(21%)
MMR	39(16%)	41(26%)
DMR	36(15%)	52(32%)
Unevaluable	82(34%)	2(1%)

CCyR, complete cytogenetic response; MMR, major molecular response; DMR, deep molecular response.

*Dasatinib dosage referred to current dose in cross-sectional cohort and initial dose in longitudinal cohort.

Data of 161 patients with CML-CP receiving dasatinib therapy and undergoing regular echocardiographic evaluation before and during dasatinib therapy established the longitudinal dataset (Table 1). The median age was 46 years (range, 11–79 years) at the start of dasatinib therapy. 100 (62%) patients were male. The median duration of CML before dasatinib therapy was 10 months (range, 0–231 months). The median duration of dasatinib therapy was 24 months (range, 3–156 months). 182 (79%) patients received dasatinib as the 2nd-line of therapy. 52 (32%) patients were treated with an initial dasatinib dose of <100 mg/d.

### In the cross-sectional cohort

Echocardiographic screening showed that the average TRV was 2.6 ± 0.5 m/s in 243 patients. Thirty (12%) patients were classified as having a high probability of PH at a median dasatinib therapy duration of 27 months (range, 3–129 months). Only three (10%) patients had symptoms of dyspnea, reduced exercise capacity, exertional syncope, or other signs of right ventricular dysfunction. The average PASP was 30.4 ± 11.5 mmHg. A total of 42 (17%) patients developed PASP ≥ 40 mmHg at a median dasatinib therapy duration of 23 months (range, 3–129 months) and only four (10%) of them had the above-mentioned symptoms. A total of 23 (10%) patients were diagnosed with pericardial effusion when screening by echocardiography, including 17 patients classified as mild, five as moderate, and one as large. A total of 69 (28%) patients had pleural effusion on chest radiography.

Patient characteristics, including sex, age, disease duration before dasatinib treatment, dasatinib treatment duration, line of dasatinib used, cardiopulmonary diseases, smoking, body surface area (BSA), current treatment response, pericardial effusion, and pleural effusion, were analyzed to identify variables associated with a high probability of PH and PASP ≥ 40 mmHg. The results of the univariate analysis are shown in Table 2. In multivariate analysis, increasing age (OR = 1.7, 95% CI 1.2–2.4, *p* = 0.002 and OR = 1.5, 95% CI 1.2–2.1, *p* = 0.003) and pericardial effusion (OR = 4.3, 95% CI:1.6–11.6, *p* = 0.004 and OR = 3.2, 95% CI:1.3–8.3, *p* = 0.014) were significantly associated with both a high probability of PH and PASP ≥ 40 mmHg.

**TABLE 2:** Univariable analysis of associated variables in cross-sectional cohort.

	PASP ≥ 40 mmHg		High probability of PH	
	OR (95%CI)	*P-value*	OR (95%CI)	*P-value*
Female (ref. male)	1.2(0.6–2.3)	0.625	0.6(0.3–1.4)	0.259
Age^a^	1.6(1.2–2.1)	0.002	1.7(1.2–2.4)	0.001
Disease duration before dasatinib-therapy^b^	1.1(1.0–1.1)	0.140	1.1(1.0–1.2)	0.195
Dasatinib-therapy duration^b^	1.1(1.0–1.2)	0.166	1.1(1.0–1.3)	0.069
Dasatinib-therapy used as		0.324		0.556
** First - line (ref.)**				
Second - line	4.5(0.6–35.1)	0.148	2.7(0.3–21.2)	0.347
Third or fourth - line	3.7(0.4–30.5)	0.231	3.2(0.4–27.2)	0.281
Pericardial effusion (ref. none)	3.6(1.5–9.1)	0.006	4.8(1.8–12.6)	0.001
Pleural effusion (ref. none)	2.2(0.9–5.2)	0.069	1.9(0.7–4.8)	0.019
Cardiopulmonary diseases (ref. none)	1.6(0.6–4.3)	0.352	2.6(0.9–7.0)	0.069
Smoke (ref. none)	1.1(0.5–2.8)	0.775	1.5(0.6–3.9)	0.439
Body surface area^c^	0.8(0.1–4.5)	0.758	2.3(0.3–16.6)	0.416
Response		0.330		0.255
**<CCyR (ref.)**				
CCyR	0.6(0.2–2.1)	0.969	1.7(0.4–6.7)	0.471
MMR	1.1(0.4–3.3)	0.300	3.3(0.9–11.7)	0.065
DMR	0.5(0.1–1.9)	0.343	1.4(0.3–5.9)	0.669

95%CI, 95% confidence interval; OR, odds ratio; PASP, pulmonary artery systolic pressure; PH, pulmonary hypertension; CCyR, complete cytogenetic response; MMR, major molecular response; DMR, deep molecular response.

^a^Linear with estimates of HRs for every increase of 10 years.

^b^Linear with estimates of HRs for every increase of 1 year.

^c^Linear with estimates of HRs for every increase of 1 m^2^.

**FIGURE 1:**
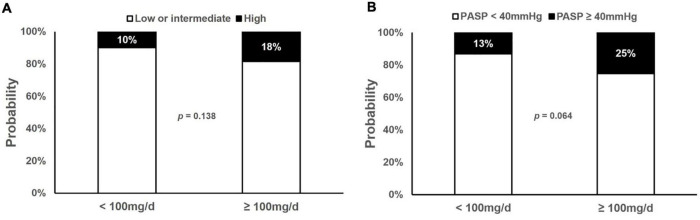
Prevalence of high probability of PH and PASP ≥ 40 mmHg. Prevalence of high probability of pulmonary hypertension (PH) and pulmonary artery systolic pressure (PASP) ≥ 40 mmHg in subjects receiving current dose < 100 mg/d and ≥ 100 mg/d dasatinib showed no significant difference. **(A)** Prevalence of high probability of PH in cross-sectional cohort. **(B)** Prevalence of PASP ≥ 40mmHg in cross-sectional cohort.

To investigate the effect of the current dasatinib dose on the development of PH, PSM analysis was performed to minimize the gap between the dasatinib doses of < 100 mg/d (*n* = 66) and ≥ 100 mg/d (*n* = 177). Because there were significant differences in the baseline characteristics between the two subgroups, covariates were matched for sex, age, internal diagnosis to initiation of dasatinib therapy, dasatinib line used in TKI therapy, BSA, and cardiopulmonary diseases (Table 3). A total of 164 patients were included, including 61 with a current dose of dasatinib < 100 mg/d and 103 with a dose ≥ 100 mg/d. There was no significant difference in the prevalence of a high probability of PH between the two subgroups (10% *vs.* 18%, *p* = 0.138); however, those with a current dasatinib dose ≥ 100 mg/d had a high likelihood of PASP ≥ 40 mmHg (13% *vs.* 25%, *p* = 0.064) (Figures 1A,B).

**TABLE 3:** Patients’ characteristics by propensity score matching analysis in cross-sectional cohort.

	<100 mg/d *N* = 61	≥100 mg/d *N* = 103	*P-*value
Male, n(%)	38(59%)	59(57%)	0.528
Age, y, median (range)	48(24–78)	47(22–72)	0.716
Body surface area, m^2^, median (range)	1.8(1.5–2.2)	1.8(1.3–2.2)	0.885
Cardiopulmonary diseases, n(%)	10(16%)	10(10%)	0.206
Time before dasatinib-therapy, m, median (range)	27(0–248)	38(0–231)	0.577
Dasatinib-therapy. used as, n(%)			0.616
First-line	8(13%)	9(9%)	
Second-line	36(59%)	67(65%)	
Third- or fourth-line	17(28%)	27(26)	

### In the longitudinal cohort

**FIGURE 2:**
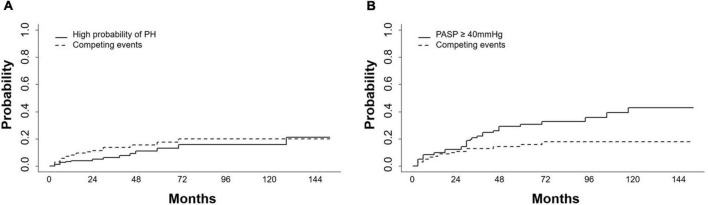
Cumulative incidences of high probability of PH and PASP ≥ 40mmHg. **(A)** Cumulative incidences of high probability of pulmonary hypertension (PH) and **(B)** pulmonary artery systolic pressure (PASP) ≥ 40mmHg increased during dasatinib-therapy. Competing events included switching to another TKI, chemotherapy, a transplant or death.

Of the 161 patients on dasatinib therapy with regular echocardiography follow-up, 109 received dasatinib at an initial dose of 100 mg/d and 52 at an initial dose of <100 mg/d (including two at 80 mg/d, two at 70 mg/d, and 48 at 50 mg/d). At the end of the observation period, 40 of 109 (37%) patients receiving the initial dasatinib dose of 100 mg/d reduced to a dasatinib dose of 20–80 mg, 6 of 52 (12%) receiving the initial dasatinib dose of 50–80 mg reduced to 20–50 mg for reasons such as favorable response (*n* = 38) or intolerance (two cases of pleural effusions and six edema), 26 switched to another TKI therapy due to dasatinib resistance (*n* = 24) or pleural effusion (*n* = 2) without any sign of PH, and one died of blast crisis during dasatinib therapy. During the follow-up period of dasatinib therapy, pericardial effusion was detected in 41 patients (25%) (35 mild and 6 moderate) at a median of 14 months (range, 0–123 months) and pleural effusion was detected in 61 patients at a median of 12 months (range, 1–79 months). Sixteen patients developed a high probability of PH at a median of 24 months (range, 3–156 months). A total of 37 patients developed PASP ≥ 40 mmHg at a median of 24 months (range, 3–156 months). The 1-, 2-, and 3-year cumulative incidences of high probability of PH were 4.8% (4.7, 4.9), 5.8% (5.7, 5.9), and 9.3% (9.1, 9.4), respectively. The 1-, 2-, 3-year cumulative incidences of PASP ≥ 40 mmHg were 9.8% (9.6, 9.9), 12.3% (12.2, 12.5) and 22.1% (21.8, 22.4), respectively (Figures 2A,B).

**FIGURE 3:**
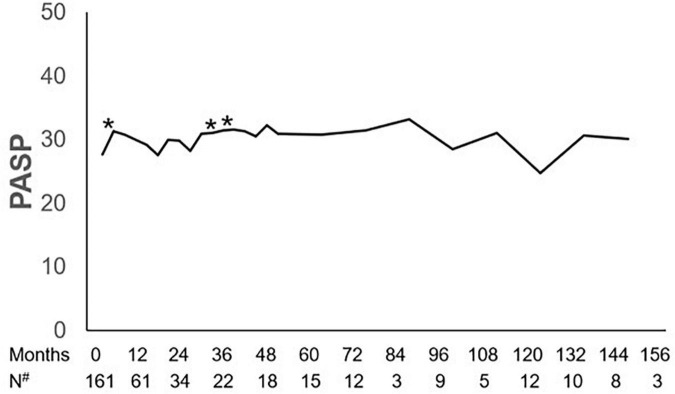
Dynamics of PASP. Pulmonary artery systolic pressure (PASP) at 3, 30, and 36 months during dasatinib-therapy was significantly higher than that at baseline. *P-value < 0.05 compared with baseline. ^#^Number of subjects who had echocardiographic data during the follow-up.

**FIGURE 4:**
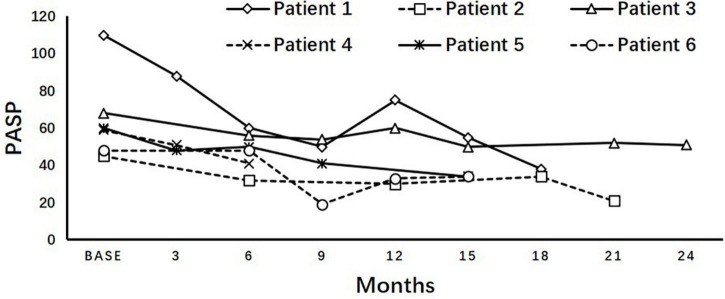
Variation of PASP after reducing dose. Longitudinal pulmonary artery systolic pressure (PASP) after reducing dasatinib dosage in asymptomatic patients diagnosed with high probability of pulmonary hypertension showed an inclination of decreasing without deteriorating the treatment response, but not all of them can fully return to <40 mmHg.

The dynamics of PASP on dasatinib therapy in the longitudinal cohort are shown with data censored at the time of reducing dasatinib dose, switch to another TKI, chemotherapy, a transplant, or death (Figure 3). In all patients, PASP was significantly higher than that at baseline at 3, 30, and 36 months. Among the 37 patients with PASP ≥ 40 mmHg, 14 received reduced dasatinib doses due to pleural effusion (*n* = 11) or dyspnea correlated with PH (*n* = 3), 9 received diuretics, and 3 received bosentan, sildenafil, or calcium channel blockers simultaneously. Finally, PASP decreased to <40 mmHg in 4 of 14 patients, and 10 switched to another TKI therapy due to persistently elevated PASP ≥ 40 mmHg (*n* = 3), pleural effusion (*n* = 3), pericardial effusion (*n* = 1), or resistance (*n* = 3). In 15 asymptomatic patients who were classified as having a high probability of PH, 7 received a reduced dasatinib dose and 5 switched to another TKI due to persistently elevated PASP ≥ 40 mmHg (*n* = 3) or resistance (*n* = 2). PASP decreased to < 40 mmHg in 3 patients when reducing dasatinib dose ± diuretics without negatively affecting the treatment response (the dynamics of PASP in 6 subjects with ≥ 3 echocardiographs after reducing dose are shown in Figure 4).

Patient characteristics, including sex, age, disease duration before dasatinib treatment, line of dasatinib use, cardiopulmonary diseases, smoking, BSA, treatment response, pericardial effusion (whether it occurred during dasatinib therapy), and pleural effusion (whether it occurred during dasatinib therapy) were analyzed to identify variables associated with a high probability of PH and PASP ≥ 40 mmHg. The results of the univariate analysis are shown in Table 4. In multivariate analysis, pericardial effusion (HR = 3.8, 1.5–9.7, *p* = 0.005) and cardiopulmonary diseases (HR = 3.2, 1.2–8.5, *p* = 0.021) were significantly associated with a high probability of PH; increasing age (HR = 1.5, 1.2–1.8, *p* < 0.001) and dasatinib as ≥ 3*^rd^*-line TKI therapy (*p* = 0.032; 2*^nd^*-line *vs.* 1^st^-line: HR = 2.0, 0.7–6.0, *p* = 0.200; ≥ 3*^rd^*-line *vs.* 1^st^-line: HR = 3.4, 1.0–11.5, *p* = 0.047) were significantly associated with PASP ≥ 40 mmHg (Table 5).

**TABLE 4:** Univariable analysis of associated variables in longitudinal cohort.

	PASP ≥ 40 mmHg		High probability of PH	
	OR (95%CI)	*P-value*	OR (95%CI)	*P-value*
Female (ref. male)	1.3(0.7–2.5)	0.393	1.0(0.4–2.5)	0.949
Age^a^	1.5(1.2–1.8)	< 0.001	1.2(0.9–1.6)	0.170
Disease duration before dasatinib-therapy^b^	1.0(0.9–1.1)	0.900	1.0(0.9–1.2)	0. 830
Dasatinib-therapy used as		0.116		0.611
**First - line (ref.)**				
Second - line	1.9(0.6–5.4)	0.260	1.0(0.3–3.7)	0.990
Third or fourth - line	3.5(1.0–11.4)	0.042	1.9(0.5–9.6)	0.420
Pericardial effusion (ref. none)	2.0(1.0–3.8)	0.039	1.3(0.2–10.9)	0.003
Pleural effusion (ref. none)	1.8(1.0–3.4)	0.081	1.9(0.7–4.8)	0.215
Cardiopulmonary diseases (ref. none)	2.4(1.2–4.6)	0.003	3.5(1.4–8.8)	0.004
Smoke (ref. none)	0.7(0.3–1.6)	0.342	0.4(0.1–2.0)	0.247
Body surface area^c^	0.6(0.1–2.6)	0.510	2.4(0.4–14.3)	0.330
Response		0.720		0.920
**< CCyR (ref.)**				
CCyR	1.4(0.5–3.6)	0.520	1.8(0.4–7.8)	0.440
MMR	1.3(0.5–3.3)	0.560	1.6(0.4–6.9)	0.510
DMR	0.9(0.4–2.3)	0.820	1.1(0.2–4.5)	0.940

95%CI, 95% confidence interval; HR, hazard ratio; PASP, pulmonary artery systolic pressure; PH, pulmonary hypertension; CCyR, complete cytogenetic response; MMR, major molecular response; DMR, deep molecular response.

^a^Linear with estimates of HRs for every increase of 10 years.

^b^Linear with estimates of HRs for every increase of 1 year.

^c^Linear with estimates of HRs for every increase of 1 m^2^.

**TABLE 5:** Multivariable analysis of associated variables in longitudinal cohort.

	PASP ≥ 40 mmHg		High probability of PH	
	OR (95%CI)	*P-value*	OR (95%CI)	*P-value*
Age^a^	1.5(1.2–1.8)	<0.001		
Dasatinib-therapy used as		0.032		
First - line (ref.)				
Second - line	2.0(0.7–6.0)	0.200		
Third or fourth - line	3.4(1.0–11.5)	0.047		
Pericardial effusion (ref. none)			3.8(1.5–9.7)	0.005
Cardiopulmonary diseases (ref. none)			3.2(1.2–8.5)	0.021

95%CI, 95% confidence interval; HR, hazard ratio; PASP, pulmonary artery systolic pressure; PH, pulmonary hypertension.

^a^Linear with estimates of HRs for every increase of 10 years.

**FIGURE 5:**
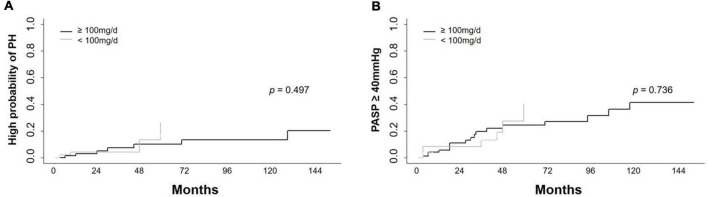
Cumulative incidence of high probability of PH and PASP ≥ 40 mmHg in different dose groups. Cumulative incidence of high probability of pulmonary hypertension (PH) and PASP ≥ 40 mmHg in subjects receiving dasatinib with mean dose < 100 mg/d and ≥ 100 mg/d showed no significant difference**. (A)** Cumulative incidence of high probability of PH in longitudinal cohort. **(B)** Cumulative incidence of PASP ≥ 40 mmHg in longitudinal cohort.

Because the dasatinib dose was changed in some patients during therapy, patients were divided into two subgroups: those receiving a mean dose of dasatinib < 100 mg/d and ≥ 100 mg/d. To explore the impact of dasatinib dose on PASP during therapy, PSM analysis was performed and the covariates were matched for sex, age, time from diagnosis to initiation of dasatinib therapy, dasatinib line used in TKI therapy, BSA, and cardiopulmonary diseases (Table 6). A total of 124 patients were matched (48 on dasatinib therapy < 100 mg/d and 76 on ≥ 100 mg/d). There was no significant statistical difference in the cumulative incidence of high probability of PH and probability of PASP ≥ 40 mmHg between the two subgroups (*p* = 0.497 and 0.736) (Figures 5A,B).

**TABLE 6:** Patients’ characteristics by propensity score matching analysis in longitudinal cohort.

	<100 mg/d *N* = 61	≥100 mg/d *N* = 103	*P-value*
Male, n(%)	29(60%)	47(62%)	0.874
Age, y, median (range)	46(11–73)	40(11–68)	0.167
Body surface area, m^2^, median (range)	1.8(1.5–2.4)	1.8(1.4–2.6)	0.807
Cardiopulmonary diseases, n(%)	14(29%)	16(21%)	0.304
Time before dasatinib-therapy, m, median (range)	8(0–180)	12(0–231)	0.914
Dasatinib-therapy. used as, n(%)			0.952
First-line	9(19%)	16(21%)	
Second-line	35(73%)	54(71%)	
Third- or fourth-line	4(8%)	6(8%)	0.874

## Discussion

In this study, the prevalence and associated variables of dasatinib-related PH were investigated, and increasing age, pericardial effusion, and cardiopulmonary diseases were significantly associated with a high probability of PH. Increasing age, pericardial effusion, and dasatinib as ≥ 3*^rd^*-line TKI therapy were significantly associated with PASP ≥ 40 mmHg.

Consistent with previous reports (6, 16), we found that increasing age was significantly associated with PH. In the current study, the age of the patients was 10–15 years younger than those of the studies from western countries (17, 18). Therefore, the actual incidence of PH may be underestimated.

In our cross-sectional and longitudinal study, we first reported that previous or simultaneous occurrence of pericardial effusion was significantly associated with a high probability of PH and PASP ≥ 40 mmHg on echocardiography in CML patients receiving dasatinib therapy. Unlike in the DASISION study, PH was usually observed concomitant with pleural effusion, which is a common AE caused by off-target effects of dasatinib likely related to the kinase inhibition of PDGFR-β or Src kinases (3). We speculated that pericardial effusion, an uncommon AE of fluid retention on dasatinib therapy, might be induced by a similar mechanism (19). Therefore, pericardial effusion could be correlated with the occurrence of PH.

We also found that dasatinib as ≥3rd line TKI therapy was significantly associated with the cumulative incidence of PASP ≥ 40 mmHg, which could indicate that previous TKI therapy had a potential impact on the onset of PH. Imatinib and nilotinib were approved as the 1^st^-line TKI in patients with CML-CP in China and were widely used as the 1st- or 2nd-line TKI therapy in the current study. Nilotinib has been reported to injure vascular endothelial cells by direct pro-atherogenic and anti-angiogenic effects and might be associated with the cumulative effect on the onset of PH (20). Consistent with previous studies, PH was more likely to occur in elderly patients who received dasatinib therapy (6, 16). Understandably, patients with cardiopulmonary comorbidities were prone to develop a high probability of PH when receiving dasatinib therapy.

The effect of dasatinib dose on PH has been a topic of debate in previous studies (21). In our cross-sectional cohort, patients with a current dasatinib dose ≥ 100 mg/d had a tendency toward a PASP ≥ 40 mmHg (*p* = 0.06), but a similar prevalence of high probability of PH as those with dose < 100 mg/d did. In the longitudinal cohort, dasatinib dose had no impact on PASP over time. A larger number of patients and longer follow-up period are needed to investigate the impact of dasatinib dose on the occurrence of PH in future studies.

This study had several limitations: (1) This was a retrospective study. (2) Initial dasatinib dose was inconsistent and adjusted because of favorable response or non-PH related AEs in some patients during treatment in the longitudinal cohort, which may influence the estimation of the PASP variation. Although switching to another therapy was considered a competing event and PSM analysis was performed, the impact of dasatinib dose on the onset of PH could not be compared perfectly. (3) Adherence was not evaluated in longitudinal subjects; the irregularity of follow-up time points resulted in dispersive data at some time points, which might have affected the evaluation of PASP dynamics. (4) There was no data of invasive right-heart catheterization which was the gold diagnostic standard of PH. (5) There was no data of BNP level which was previously reported to be associated with dasatinib-induced PH (6).

## Conclusion

Pericardial effusion, cardiopulmonary comorbidities, and dasatinib administration as ≥ 3rd-line TKI therapy, with the exception of increasing age, were associated with the onset of PH determined by echocardiography in patients on dasatinib therapy. Our study may help physicians make treatment decisions and identify high-risk patients for dasatinib-related PH.

## Data availability statement

The original contributions presented in this study are included in the article/supplementary material, further inquiries can be directed to the corresponding authors.

## Ethics statement

The studies involving human participants were reviewed and approved by the Ethics Committee of Peking University People’s Hospital (Ethical Approval Number: 2019PHB203-01). The patients/participants provided their written informed consent to participate in this study.

## Author contributions

QJ and TZ designed the study. WJ, SY, and CY collected and analyzed the data. WJ, SY, and QJ prepared the typescript. All authors approved the final typescript, take responsibility for the content and agreed to submit for publication.
